# Delphi panel to obtain clinical consensus about using long-acting injectable antipsychotics to treat first-episode and early-phase schizophrenia: treatment goals and approaches to functional recovery

**DOI:** 10.1186/s12888-023-04928-0

**Published:** 2023-06-21

**Authors:** Celso Arango, Andrea Fagiolini, Philip Gorwood, John M. Kane, Sergio Diaz-Mendoza, Navdeep Sahota, Christoph U. Correll

**Affiliations:** 1 Child and Adolescent Department of Psychiatry, Institute of Psychiatry and Mental Health, IiSGM, CIBERSAM, School of Medicine, Hospital General Universitario Gregorio Marañón, Universidad Complutense, Madrid, Spain; 2 Department of Molecular and Developmental Medicine, Division of Psychiatry, Universita di Siena, Siena, Italy; 3 Université Paris Cité, INSERM U1266, GHU Paris Psychiatrie et Neurosciences (CMME), Paris, France; 4 Institute of Behavioral Science, Feinstein Institutes for Medical Research–Northwell Health, New York, NY USA; 5 Department of Psychiatry, The Zucker Hillside Hospital, Northwell Health, Glen Oaks, NY USA; 6 OPEN Health, Patient-Centered Outcomes, Marlow, UK; 7 OPEN Health, Patient-Centered Outcomes, London, UK; 8 Department of Psychiatry and Molecular Medicine, Donald and Barbara Zucker School of Medicine at Hofstra/Northwell-Hempstead, New York, NY USA; 9 Department of Child and Adolescent Psychiatry, Charité - Universitätsmedizin Berlin, Berlin, Germany

**Keywords:** Schizophrenia, Antipsychotics, Long-acting injectable, First episode psychosis, Early-phase schizophrenia, Functional recovery, Delphi process, Recommendations, Clinical consensus

## Abstract

**Background:**

Schizophrenia is mostly a chronic disorder whose symptoms include psychosis, negative symptoms and cognitive dysfunction. Poor adherence is common and related relapse can impair outcomes. Long-acting injectable antipsychotics (LAIs) may promote treatment adherence and decrease the likelihood of relapse and rehospitalization. Using LAIs in first-episode psychosis (FEP) and early-phase (EP) schizophrenia patients could benefit them, yet LAIs have traditionally been reserved for chronic patients.

**Methods:**

A three-step modified Delphi panel process was used to obtain expert consensus on using LAIs with FEP and EP schizophrenia patients. A literature review and input from a steering committee of five experts in psychiatry were used to develop statements about patient population, adverse event management, and functional recovery. Recruited Delphi process psychiatrists rated the extent of their agreement with the statements over three rounds (Round 1: paper survey, 1:1 interview; Rounds 2–3: email survey). Analysis rules determined whether a statement progressed to the next round and the level of agreement deemed consensus. Measures of central tendency (mode, mean) and variability (interquartile range) were reported back to help panelists assess their previous responses in the context of those of the overall group.

**Results:**

The Delphi panelists were 17 psychiatrists experienced in treating schizophrenia with LAIs, practicing in seven countries (France, Italy, US, Germany, Spain, Denmark, UK). Panelists were presented with 73 statements spanning three categories: patient population; medication dosage, management, and adverse events; and functional recovery domains and assessment. Fifty-five statements achieved ≥ 80% agreement (considered consensus). Statements with low agreement (40-79%) or very low agreement (< 39%) concerned initiating dosage in FEP and EP patients, and managing loss of efficacy and breakthrough episodes, reflecting current evidence gaps. The panel emphasized benefits of LAIs in FEP and EP patients, with consensus that LAIs can decrease the risk of relapse, rehospitalization, and functional dysfunction. The panel supported links between these benefits and multidimensional longer-term functional recovery beyond symptomatic remission.

**Conclusions:**

Findings from this Delphi panel support the use of LAIs in FEP and EP schizophrenia patients regardless of disease severity, number of relapses, or social support status. Gaps in clinician knowledge make generating evidence on using LAIs in FEP and EP patients critical.

## Introduction

Schizophrenia is one of the most burdensome and costly illnesses worldwide due to its common onset in adolescence or early adulthood and its high rate of disability [1]. Symptoms of psychosis, such as hallucinations, delusions, and disordered behavior and speech [2, 3], can affect all areas of the patient’s life, including personal, familial, social, educational, and occupational functioning [3]. Furthermore, family members can suffer as a result of the shifting of care from hospital to families [4].

Antipsychotic medication is the primary modality for the treatment of schizophrenia and should not be delayed [3]. When diagnosis and treatment are delayed, patients are at greater risk for poorer outcomes, such as a lesser response to treatment and discontinuation of it, which can exacerbate illness and increase chances of relapses.[5] Rates of non-adherence may be higher for schizophrenia patients than for patients with other chronic illnesses due to adverse events, a lack of efficacy, challenges in symptom control, poor insight into illness, cognitive dysfunction, substance abuse, stigma, and social drift [6–8]. Poor adherence to antipsychotic medication can lead to relapse and rehospitalization, as well as functional decline [9, 10].

The goals of schizophrenia management are not only symptom reduction in the short-term, but also relapse prevention, maintenance of physical and mental well-being, improved health related quality of life (HRQoL), and full functional recovery [11, 12]. Long-acting injectable antipsychotics (LAIs) have been historically used for schizophrenia patients, who have exhibited non-adherence; however, there is evidence that they are potentially beneficial for all patients with schizophrenia, as they improve treatment adherence, decrease treatment discontinuation, and may reduce the risk of relapse and rehospitalization where there have still been reports of patients experiencing breakthrough psychotic events despite continuous (oral) treatment [9, 13, 14]. In addition, use of LAIs can improve adherence to medications addressing cardiometabolic risk, facilitate functioning, and decrease all-cause as well as specific-cause mortality [5, 6, 12, 14–17]. However, LAIs are frequently underused in current practice, with a highly heterogeneous pattern of use among countries due to perceived stigma as being coercive, service barriers, and lack of clinician knowledge [5, 18, 19].

Given that the early illness phase of schizophrenia is a high-risk period for treatment non-adherence, related relapse and increased mortality compared to age-matched individuals from the general population and that LAIs are underutilized in this population despite being shown to significantly decrease those risks in that same population in real-world data sets, there is a knowledge gap regarding potential barriers to earlier use of LAIs in the treatment algorithm and ways to overcome them [5, 14, 15, 17, 20, 21, 29]. Without addressing this knowledge gap from a clinical implementation angle, opportunities for positively influencing the early illness course of individuals with schizophrenia at an illness stage where they are closest to their premorbid social and educational networks will be missed.

Because LAIs are typically regarded as a last resort, they are usually reserved for more seriously ill, chronic patients. However, they may benefit first-episode psychosis (FEP) or early-phase (EP) patients, who arguably have the most to gain if treated early, and the most to lose if not, as use of these formulations can prevent relapses, and functional decline [19]. Aside from describing their use in patients with obvious non-adherence, the literature on the clinical benefits of LAIs is sparse, and their benefits in clinical populations, such as early-stage schizophrenia patients or those without multiple hospitalizations, should be explored [20, 21].

To explore some of the underlying aspects of underutilization and to better evaluate the potential benefits of using LAIs in FEP and EP schizophrenia patients, we aimed to obtain expert consensus on the use of LAIs for the treatment of patients with FEP or EP schizophrenia. For this study, the definition of EP schizophrenia was adapted from that used by the American Psychiatric Association [22] and given as the period after recovery from a first episode of schizophrenia and extending up to the subsequent three years. We did not distinguish between FEP and EP, but by definition, all would be FEP, and some will have more than one episode of psychosis and be defined as EP in those first three years.

## Methods

### Study design

**Fig. 1:**
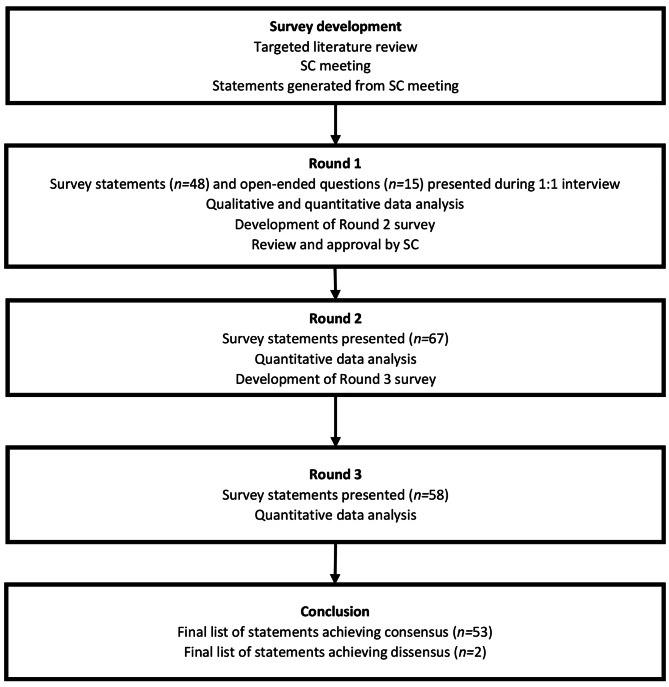
Modified Delphi framework

A Delphi panel is an iterative technique characterized by repeated rounds of controlled feedback until consensus is achieved; in this manner, it allows the systematic collection and aggregation of informed judgments from experts [23, 24]. To meet the study objective, a three-step modified Delphi technique was used consisting of a single 1:1 interview round and two survey rounds (Fig. 1). The first round survey was developed from a targeted literature review and discussion with a steering committee (SC) rather than from an initial open-ended round of statements as would happen with a classical Delphi panel [25]. Five psychiatrists (CA, AF, PG, JK, CC) with expertise in schizophrenia and LAIs formed the SC, providing input into the study design, potential panelists, and survey development.

### Panelist selection

To reach the target sample of 18, as per the recommended sample size of 5–20 individuals [26], the SC were asked to recommend Delphi panelists from the United States (US), United Kingdom (UK) and Europe.

Panelists were screened according to the following inclusion criteria:

- Practicing psychiatrist.
- Seen a minimum of 10 patients with schizophrenia in the last two years.
- Has published in the area of schizophrenia.

Forty-two clinicians were invited by email to participate in the Delphi panel (38 recommendations from the SC, and four from the study Sponsors who are known for their work in the schizophrenia disease space including the development of LAIs). Twenty clinicians accepted the invitation, and 17 took part in the Round 1 interview.

### Preparation

A meeting was held with the SC in February 2021 with the following agenda:

- Outline of the Delphi Panel statement framework (developed from a targeted literature review including guidelines for LAI use, naturalistic prospective studies, non-interventional clinical trials, systematic literature reviews, and randomized controlled trials).
- Discussion of analysis rules for consensus statements.
- Suggestion of additional experts to participate as panelists.

It was agreed to use the following sections to address the objective with subsections added after initial review of the final dataset (Table 1):

**Table 1:** Statement categorization

Section 1: Patient population	Section 2: Dosage, management & adverse events	Section 3: Functional recovery domains & assessment
• Generic LAI use • Potential exclusions • Appropriateness for FEP and EP patients	• Adverse event management • Psychotic episodes on LAI • Loss of efficacy • LAI dosing & intervals for FEP and EP patients	• Long-term treatment goals • LAI links to functional recovery • Functional recovery domain • Functional recovery assessment • Achieved functional recovery

### Procedure

The Delphi panel was conducted between April and November 2021. Potential panelists were invited via email to participate. Written consent was obtained from those invited who agreed to participate, and their Round 1 interviews scheduled. Round 2 and 3 surveys were emailed to panelists, who were typically given 14 days to return their responses (reminders were sent at regular intervals).

### Survey development

During each of the three rounds, panelists were asked to rate the extent to which they agreed with each statement using either Likert scales or binary responses. In Round 1, all statements were presented with a 5-point Likert response scale (1 = Completely Disagree, 2 = Disagree, 3 = Neutral, 4 = Agree, 5 = Completely Agree). Open-ended questions were asked only during the first round to generate further statements for Round 2. Areas for comments under each statement were also incorporated, allowing panelists to provide additional qualitative insights. After the first round, 3-point Likert scale (1 = Disagree, 2 = Neutral, 3 = Agree) or binary response options (Disagree, Agree) were selected by the researchers and approved by the SC depending on the percentage frequencies a statement achieved in the prior round, as per the analysis rules (Table 2).

### Round 1

The Round 1 (April–July 2021) survey was completed by panelists during a 1:1 audio-recorded teleconference interview with a researcher, to facilitate discussion of the statements. Panelists were provided a structured list of 40 statements and 15 open-ended questions to collect both quantitative and qualitative data. Panelists’ qualitative data were used to generate further statements for the Round 2 survey (Fig. 1).

### Rounds 2 and 3

According to the modified Delphi methodology, all open-ended questions and the three statements that did not achieve the minimum response threshold (41%) during Round 1 were removed from the survey. Twenty-two new statements were generated following analysis of panelists’ qualitative responses to the open-ended questions and their comments on pre-existing statements, resulting in a 67-item Round 2 survey.

The Round 2 survey was customized for each panelist, presenting the panelist’s individual responses and the group mode, mean, and interquartile range (IQR) for statements brought forward from Round 1. Round 2 was conducted between August and September 2021. Following quantitative analysis of the Round 2 survey data, nine statements were removed (seven achieving consensus, two not achieving the minimum response threshold) as per the analysis rules, leaving 58 items in the Round 3 survey (Fig. 1). Similar to Round 2, individual and group responses were reported to panelists in the Round 3 survey, which was sent to panelists in October 2021.

### Data analysis and definition of consensus

Qualitative comments and answers from the panelists’ Round 1 interview were reviewed and addressed either to refine existing statements or to create new statements for the Round 2 survey. After each round, quantitative survey responses were extracted for each statement into a Microsoft Excel database and were assigned a score/code (i.e., 1–5, 1–3, or 1, 2) corresponding to the appropriate Likert or binary response scale. The IQR was calculated and used to summarize the extent of the spread of the data. Central tendencies (mean, median, and mode) were calculated to present the group’s responses back to panelists, and percentage response frequencies for each statement were calculated to determine whether consensus had been achieved. The consensus definition was determined a priori with the SC and was later refined and standardized into the following set of analysis rules (Table 2).

**Table 2:** Analysis rules

Rule 1: Questions that show a variable response pattern (≤ 40%) spread across response options in a non-skewed way will be removed
Rule 2: Questions with responses between 41% and 79% will be re-asked with three response options: Disagree, Neutral, Agree
Rule 3: Questions that showed a skewed response pattern, with the majority of responses (≥ 80%) spread across 5 or 3 options, will be summed and asked back with a binary response option: Agree or Disagree
Rule 4: Binary questions that showed a response pattern of ≥ 80% agreement will be considered consensus
Rule 5: 3-point Likert scale questions in Round 2 with responses between 41% and 79% will be re-asked on a 3-point Likert scale in Round 3
Rule 6: 3-point Likert scale questions in Round 3 with ≥ 80% of a response option will be considered consensus

## Results

### Participation in the survey

Out of the 42 invited,17 clinicians based in seven countries (Italy, France, US, Germany, Spain, UK, Denmark) accepted the invitation to participate. All 17 participated in Rounds 1 and 2, and 16 completed Round 3.

### Overview of results

**Fig. 2:**
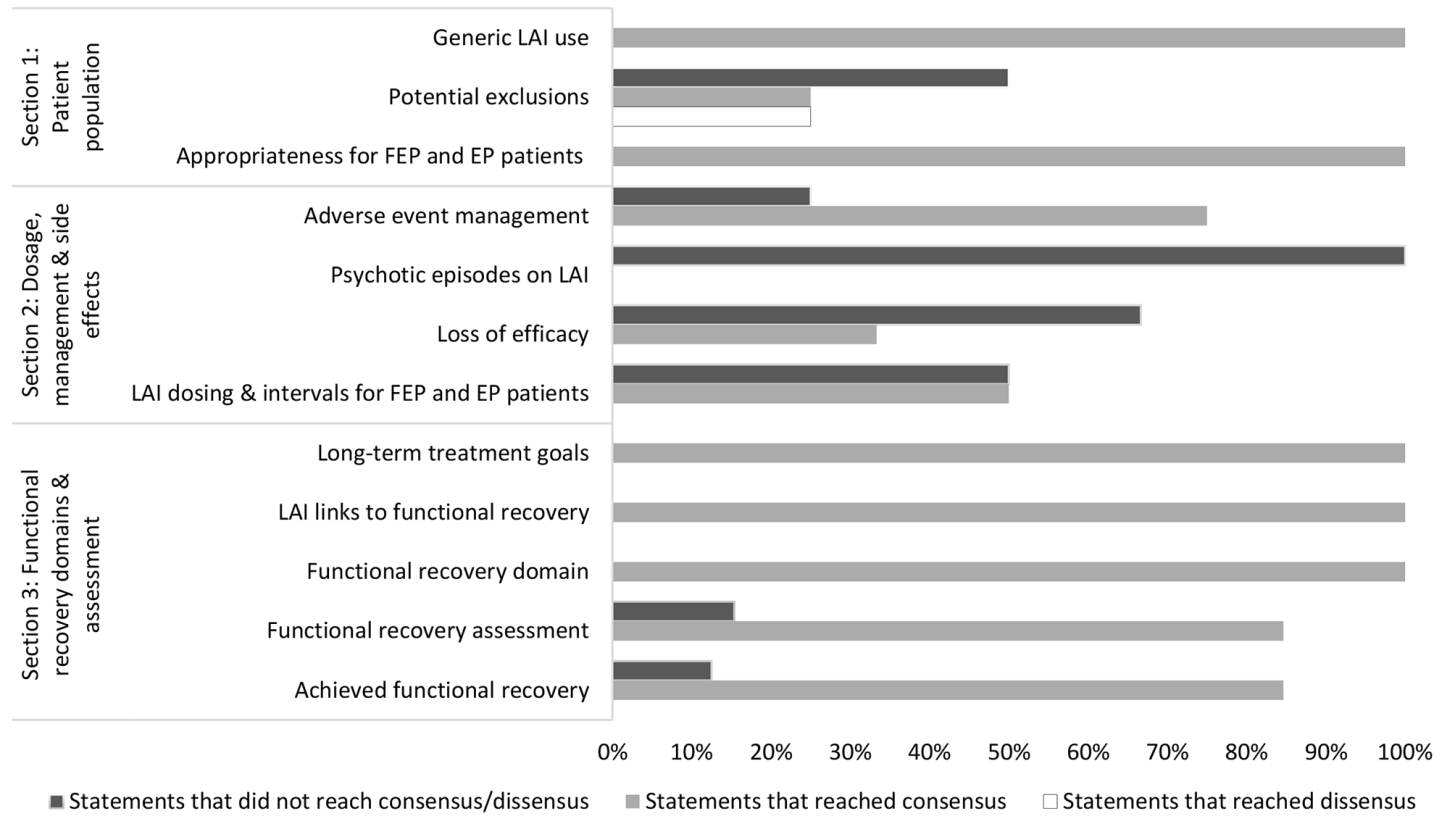
Proportion of statements per category that achieved consensus, dissensus, or neither

Overall, panelists were presented with 70 statements over three rounds. A total of 53 statements reached the minimum level of agreement (≥ 80%) to be considered a consensus. Two statements reached the minimum level of disagreement (≥ 80%) to be considered a dissensus (Fig. 2; Table 3).

**Table 3:** Top areas of consensus and dissensus among panelists

Top consensus topics	Top dissensus topics
Section 1 – Patient population	
• Generic LAI use • Appropriateness for FEP and EP patients	• Potential exclusions
**Section 2 – Dosage, management & adverse events**	
• Adverse event management	
**Section 3 – Functional recovery domains & assessment**	
• Long-term treatment goals • LAI links to functional recovery • Functional recovery domain	

### Section 1: patient population

#### Generic LAI use

In the first section, four statements were presented to the panel on generic LAI use in schizophrenia patients, of which 100% reached consensus. All patients with schizophrenia should be evaluated clinically to be considered for LAI treatment. The treatment goals and clinical management for schizophrenia patients do not differ whether they are treated with LAI or oral antipsychotic (AP) medication. Clinical management for LAIs does not differentiate between FEP and EP patients. When using LAIs, adverse events, stability, and the therapeutic alliance should be taken into consideration.

### Potential exclusions

Eight statements on potential reasons to exclude FEP or EP patients for LAI treatment were presented to the panel in Round 1, and some additional exclusions were added in Round 2 following the qualitative analysis of open-ended questions. The panel reached dissensus (≥ 80% disagree) on 25% of statements in this section: (1) a history of drug abuse and (2) patient obesity was not considered reasons to exclude FEP or EP patients from LAI treatment. There was a consensus on 25% of statements: (3) LAI treatment can be used for breastfeeding FEP and EP patients if the risk vs. benefit is carefully considered, and (4) if they are monitored regularly. There was low agreement (40-79% agreement) on 50% of statements: (5) FEP and EP patients should avoid breastfeeding while on oral or LAI treatment. Panelists did not reach consensus (≤ 39% agreement) on whether (6) pregnancy, (7) needle phobia and (8) known cardiac issues were reasons to exclude a FEP or EP schizophrenia patient from LAIs.

### Appropriateness of LAIs for FEP or EP patients

The panel were presented six statements in this section, with 100% reaching consensus.

LAI treatment is appropriate for even those FEP and EP patients who: (1) have a high level of insight into their illness, (2) have good social support, (3) are currently adherent to medication, (4) and have not had a relapse.

Panelists also reached consensus that 5) FEP or EP patients should be fully informed before switching to LAI from oral AP, and 6) it is appropriate to switch when tolerability and efficacy to the same oral AP are established.

### Section 2: dosage, management & adverse events

#### Adverse event management

The four statements in this subsection were developed from the Round 1 open-ended questions regarding steps to take if an FEP or EP patient has adverse events in the first half of the injection interval (within two weeks of a four-week interval).

75% of the statements reached consensus: the treating psychiatrist should (1) prescribe a counteracting medication depending on the adverse event, (2) observe to see if the adverse event resolves, unless urgent action is required, and (3) decrease the dose at the next interval if the adverse event does not resolve, depending on severity.

There was low agreement that 4) the use of LAIs in FEP or EP patients required any specific monitoring compared with the same or other oral APs.

### Psychotic episodes on LAI

The three statements in this subsection were developed from qualitative analysis in Round 1 on how to manage an FEP or EP patient who has a breakthrough episode while on an LAI.

No statements reached consensus on actions to take when a psychotic episode occurs when the patient is on an LAI: (1) raising the dose without switching to a different drug by adding the oral version of that LAI (depending on the current dose), (2) increasing the dose of the LAI at the next injection interval if the psychosis improves after adding the oral version of the LAI, or (3) switching to a new dose of a different oral AP and commencing with the LAI once efficacy and tolerability are established.

### Loss of efficacy

Three statements on actions to take in the case of the LAI losing efficacy were developed for Round 1 and rephrased for round 2 following feedback from both Delphi panelists and the SC.

Consensus was reached for one statement (33%): 1) if LAI treatment starts to lose efficacy for a FEP or EP schizophrenia patient, the dose can be increased depending on what dose is already being used and after ruling out other factors, such as drug use and medical or psychiatric comorbidities.

Panelists had low agreement that 2) the injection interval can be decreased when loss of efficacy occurs; 3) changing the injection site from gluteal to deltoid injections if LAI treatment starts to lose efficacy for a FEP or EP schizophrenia patient did not reach consensus.

### LAI dosing & intervals for FEP and EP patients

Panelists reviewed four statements designed to address potential concerns physicians may have using LAIs in FEP or EP patients.

The panel reached a consensus on 50% of statements: (1) that FEP and EP patients can be on the equivalent dose of their oral AP providing they are monitored for the first three months and (2) in order to establish tolerability and efficacy when switching from oral to LAIs, panelists agreed that they would use different doses of LAIs in FEP and EP patients.

There was low agreement that 3) FEP and EP patients need to commence LAIs at a lower dose because they are younger and have less medication exposure and that 4) for a FEP or EP schizophrenia patient, a monthly injection interval would be used.

### Section 3: functional recovery domains & assessment

#### Long-term treatment goals

Panelists were presented with a definition of functional recovery to ensure they were all aligned. The definition highlighted that it goes beyond symptomatic remission and encompasses multiple aspects of the patient’s life.

All five statements in this section reached consensus: (1) functional recovery is multidimensional, exists on a spectrum, and is not a binary state for a FEP or EP schizophrenia patient; (2) the assessment for functional recovery in FEP or EP schizophrenia patients involves both the use of patient-specific tools (patient-reported outcomes) in addition to (3) conversations with the patient, informants, and the clinical team; (4) functional recovery is a reachable treatment goal for schizophrenia patients, particularly if treated with medication during the FEP or EP; and (5) the patient’s attitude toward treatment and patient psychoeducation are important to achieve functional recovery in FEP or EP schizophrenia patients.

### LAI links to functional recovery

Three statements linking the use of LAIs in FEP and EP patients were presented to the panel over the three rounds, which all achieved consensus.

The use of LAIs results in a better treatment outcome and promotes functional recovery by (1) increasing adherence, (2) reducing treatment burden, and (3) reducing functional decline.

### Functional recovery domain

Nine statements about the dimensions of functional recovery were developed during the first round from the literature and SC input, with 100% reaching consensus. The following dimensions are important to assess when aiming for functional recovery in FEP and EP patients: (1) depression, (2) aggressive behavior, (3) social interaction, (4) family functioning, (5) education and/or employment, (6) sexual functioning, (7) leisure activities, and (8) self-care. In addition to the specific domains, (9) functional recovery should include an element that is meaningful to the FEP or EP schizophrenia patient. Qualitative analysis in Round 1 did not yield additional statements to be used in subsequent rounds.

### Functional recovery assessment

Thirteen statements on aspects of assessing FEP and EP patients for functional recovery were presented to the Delphi panelists, 85% reaching consensus. They were that (1) functional recovery is multidimensional, operating on a spectrum and not a binary state for a patient with FEP or EP schizophrenia; (2) the consideration of specific dimensions (e.g., depression, social functioning) is useful when assessing the extent of functional recovery in FEP or EP schizophrenia patients; (3) as a part of the assessment for functional recovery in FEP or EP schizophrenia, patient-specific tools (e.g., patient-reported outcomes) can be used; (4) conversations with the patient, informants, and the clinical team can be used as a part of the assessment for functional recovery in FEP or EP schizophrenia patients; and (5) a FEP or EP schizophrenia patient can have partial functional recovery if some of the dimensions (e.g., depression, social interaction) are either not improved or only partially improved.

In the first round, the panelists were asked to provide an example of a question they would ask about each dimension to initiate a conversation with the patient about it. In the following round, panelists were asked if they would discuss each dimension of functional recovery at every encounter with the FEP or EP patient. Panelists reached consensus they would ask about 6) depression, 7) aggressive behavior, 8) social interaction, 9) family functioning, 10) education and/or employment, and 11) self-care at every encounter. There was a low level of agreement on 15% of statements; panelists did not reach a consensus on asking about 12) leisure activities or 13) sexual functioning at every encounter with the patient.

### Achieved functional recovery

There was consensus on 85% of the 8 statements in this section: the dimensions (1) depression, (2) aggressive behavior, (3) social interaction, (4) family functioning, (5) education and/or employment, (6) leisure activities, and (7) self-care should be minimally impaired to consider the FEP or EP patient as having achieved functional recovery. Panelists did not meet the minimum threshold for consensus on the statement that (8) sexual functioning should be only minimally impaired to consider the patient to have attained functional recovery.

## Discussion

Delphi panels are widely used in healthcare research and are proven to be a rigorous and feasible way to obtain consensus, allowing for anonymous, expert input through several rounds of controlled feedback. Using a modified Delphi panel, we obtained expert consensus about using LAIs to treat patients with FEP or EP schizophrenia and promote functional recovery, yielding the following main results: (1) 55 statements achieved consensus (i.e., ≥ 80% agreement); (2) Statements with low agreement (40-79%) or very low agreement (< 39%) concerned antipsychotic initiation dosage in FEP and EP patients, and managing loss of efficacy and breakthrough episodes; (3) benefits of LAIs in FEP and EP patients include decreasing the risk of relapse, rehospitalization, and functional dysfunction, supporting the use of LAIs in FEP and EP schizophrenia patients regardless of disease severity, number of relapses, or social support status; and (4) links between these benefits and multidimensional longer-term functional recovery beyond symptomatic remission were supported.

### Current treatment guidelines

Several, but not all, current treatment guidelines suggest that LAIs should be reserved for patients who have had multiple relapses, have exhibited non-adherence to oral or LAIs in the past, have poor social support, and have previous LAI experience [12, 19]. While there is support from some guidelines, others are neutral or silent on LAI use in FEP and EP patients, or indeed some recommend against use in this population [12]. FEP patients are unlikely to have experienced a relapse, and it is not clear whether non-adherence is going to be an issue at this stage; thus, in current clinical practice, they are rarely considered for LAI treatment [20]. However, some research shows that there is evidence that non-adherence is the biggest predictive factor of relapse after a first episode of psychosis [13, 27]. Also, in FEP patients, discontinuation of antipsychotics for two or more months has been shown to significantly increase the risk of relapse, and that relapse can be prevented for up to 24 months by maintaining antipsychotic treatment in these patients [28]. This expert panel agreed to statements pertaining to the appropriateness of LAIs for all patients with schizophrenia, including FEP and EP patients. This recommendation is despite the fact that those patients’ have not yet experienced the other issues that more chronically ill, multi-episode patients may have who are usually considered for LAIs, such as poor social support. Further support for the use of LAIs was demonstrated, as the panel found that other than severe adverse events on the same oral AP that the LAI would be considered as, no other contraindications to starting patients on LAIs exist, providing they are appropriately monitored. This finding is supported by recent evidence that there is not any greater risk with LAIs compared to oral APs for the potentially fatal reaction neuroleptic malignant syndrome [29–31].

### Gaps in clinician knowledge

The lack of published guidance on how to initiate and maintain FEP and EP schizophrenia patients with LAIs has uncovered gaps in knowledge and concerns for clinicians using LAIs in this patient population [5, 18, 20]. To address these barriers, this Delphi panel indicated expert agreement that treatment goals and clinical management remain the same across patient subgroups and oral vs. LAIs. However, whether there should be specific monitoring for FEP or EP patients on LAIs compared to the same oral AP was not determined in this panel. This finding indicates that the difference in formulation of the AP does not affect general recommendations about strategies for monitoring efficacy and safety, nor is there any specific difference in monitoring requirements between FEP/EP and chronic patients.

Most statements on managing adverse events in the first half of the injection interval achieved consensus, as did statements about establishing and maintaining efficacy. Feedback from the panelists suggested that reducing the injection interval to manage insufficient/loss of efficacy is an off-label strategy or against country-specific guidance. Incorporating advice on the management of adverse events and inefficacy for FEP and EP patients on LAIs into current guidelines could still be beneficial to support less experienced clinicians, particularly considering different countries’ regulations.

While statements describing raising the dose by adding the oral version of the AP and then implementing a higher dose to counteract loss of efficacy at the next interval received moderate agreement (69% and 75%, respectively), the panelists remained overall neutral (50% agreement) regarding switching to a new oral AP and commencing LAIs once tolerability and efficacy are established as the next strategy. Additionally, statements on initiating FEP or EP patients on a lower LAI dose than for chronic patients received a low consensus. This lack of consensus reflects the fact that schizophrenia and its clinical manifestations are heterogeneous, such that no simple categorization is possible; some patients with chronic illness may only tolerate or respond to lower AP doses whereas some FEP patients may require and tolerate higher doses.

### Barriers to LAIs

Barriers to LAIs include the stigma associated with injections (being possibly perceived as coercive) and the sense that LAIs are to be reserved for patients with severe illness [19]. Many FEP and EP patients are unaware that LAIs are an option for them; however, if they are included in guidelines and patients are informed of the benefits, this could increase acceptance [5, 9, 30, 32]. There is recent evidence to suggest that some FEP and EP patients are open to LAI treatment and may even prefer it [29]. Offering LAIs as an option and eliciting patient’s preferences before switching to a specific antipsychotic agent could encourage patients and their families to consider the LAI treatment option. Additionally, a recent Delphi panel also found that experts agreed that LAIs can reduce the stigma of having to take daily medication [20].

### Long-term functional recovery

Functional recovery is a complex concept, and there remains a lack of clarity on its definition [34]. However, in this Delphi panel, recovery was conceptualized as going beyond symptomatic remission and encompassing multiple aspects of a patient’s life [35, 36]. There is evidence that functional outcomes are especially improved with LAIs, particularly when offered earlier rather than later in the illness [37–39]. This finding is supported by the results from this current Delphi panel, which attained the agreement among experts that the known benefits of LAIs (increasing adherence and reducing functional decline, rehospitalization, and treatment burden) lead to a better long-term treatment outcomes and fuller functional recovery, which has been implied in other research [9, 19, 20, 33].

Because LAIs need to be administered in a patient care setting, such as a clinic, the patient may be seen more frequently by clinicians than patients not treated with LAIs [5]. In a clinic, it is easier to evaluate how the patient is progressing towards functional recovery. The expert panel in this Delphi reached consensus that functional recovery and HRQoL are often linked, which has been further supported, for example by a long-term trial follow-up for schizophrenia patients on the LAI aripiprazole once monthly [40].

### Functional recovery approach

While there are existing scales to assess functional remission, symptom improvement, and HRQoL (e.g., Functional Remission of General Schizophrenia, Positive and Negative Syndrome Scale, and Quality of Life Scale), this current Delphi panel sought expert consensus on an approach to functional recovery. Less than full functional recovery in some domains (sexual functioning and leisure activities) is considered acceptable. Furthermore, there are aspects of functional recovery that should be individualized, to take account of the patient’s personal goals and aspirations, attitude toward treatment, and receipt of appropriate psychoeducation [36].

### Limitations

Limitations of the current study include the uneven distribution of Delphi panelists across countries, such that the group was Euro-centric. Thus, these results may not be fully generalizable to other locales. Additionally, further information on the demographics of the panelists could have been captured (e.g., years in practice), allowing for a better understanding of the effect of different experiences/expertise among panelists. Another limitation is the lack of a pilot study to further affirm the compressibility of the questionnaire and usefulness of the response options [41]. The lack of a pilot study was mitigated by the 1:1 interview in the first round to determine comprehensibility and to clarify any statements. Finally, the response option “I don’t know,” which has been used in other Delphi panels [42], was not used in this current study, which could have skewed the results.

### Recommendation

Given the potential benefits of LAIs in these population of patients, it would be prudent to incorporate LAIs into a system of integrated care which would include other psychological and psychosocial interventions such as family interventions or cognitive behavioural therapy as a way of preventing relapse and achieving functional recovery [43, 44].

### Summary and conclusion

In summary, this Delphi consensus panel regarding the potential value of LAIs for treating patients with FEP or EP schizophrenia, with a particular focus on functional recovery identified many areas of broad consensus, as well as areas with low or very low agreement, which concerned antipsychotic initiation dosage in FEP and EP patients, and best managing practices when facing inefficacy and breakthrough episodes. However, there was broad consensus that FEP and EP patients could benefit from LAIs regarding decreasing the risk of relapse, rehospitalization, and functional dysfunction, supporting the use of LAIs in FEP and EP schizophrenia patients regardless of disease severity, number of relapses, or social support status, ultimately improving opportunities for achieving multidimensional functional recovery.

## Data Availability

The datasets used and/or analysed during the current study available from the corresponding author on reasonable request.

## Abbreviations

EP: Early phase
FEP: First episode
LAI: Long-acting injectable antipsychotic
HRQoL: Health-related Quality of life
SC: Steering committee
